# CAR-T cell therapy and reconstructive oncologic surgery in peripheral solid tumors—A narrative review

**DOI:** 10.1016/j.xcrm.2025.102240

**Published:** 2025-07-16

**Authors:** Leonard Knoedler, Konstantin Herfeld, Daniel A. Schaefer, Fortunay Diatta, James Clune, Brogan Evans, Michelle Seu, Bong-Sung Kim, Michael Alfertshofer, Thomas Schaschinger, Jasper Iske, Samuel Knoedler, Alexandre G. Lellouch, Maxime Jeljeli, Alberto Carturan, Marco Ruella, Max Heiland, Hendrik Poeck, Markus Perl, Bohdan Pomahac, Martin Kauke-Navarro

**Affiliations:** 1 Division of Plastic Surgery, Department of Surgery, Yale New Haven Hospital, Yale School of Medicine, New Haven, CT, USA; 2 Charité – Universitätsmedizin Berlin, Corporate Member of Freie Universität Berlin, Humboldt-Universität zu Berlin, and Berlin Institute of Health, Department of Oral and Maxillofacial Surgery, Berlin, Germany; 3 Division of Plastic and Reconstructive Surgery, Cedars-Sinai Medical Center, Los Angeles, CA, USA; 4 Department of Internal Medicine III, Hematology and Oncology, University Hospital Regensburg, Regensburg, Germany; 5 Department of Pediatrics, Dr. von Hauner Children’s Hospital, University Hospital, LMU Munich, Munich, Germany; 6 Department of Neurology, Loyola University Medical Center, Maywood, IL, USA; 7 Department of Plastic Surgery and Hand Surgery, Zurich University Hospital, Zurich, Switzerland; 8 Department of Cardiothoracic Surgery, Deutsches Herzzentrum der Charité, Berlin, Germany; 9 Berlin Institute of Health at Charité – Universitätsmedizin Berlin, Berlin, Germany; 10 Center for Cellular Immunotherapies and Cellular Therapy and Transplant, University of Pennsylvania, Philadelphia, PA, USA; 11 Division of Hematology-Oncology, Hospital of the University of Pennsylvania, Philadelphia, PA, USA; 12 Bavarian Cancer Research Center (BZKF), Munich, Germany; 13 Leibniz Institute for Immunotherapy (LIT), Regensburg, Germany

**Keywords:** breast cancer, sarcoma, head and neck cancer, skin cancer, malignant melanoma, CAR-T cells, chimeric antigen receptor, immunotherapy, clinical trials, reconstructive surgery

## Abstract

This review addresses the integration of chimeric antigen receptor (CAR)-T cell therapy with reconstructive oncologic surgery in treating peripheral solid tumors, including melanoma, sarcomas, breast cancer, and head and neck cancers. While CAR-T cells have demonstrated effectiveness in blood cancers, their efficacy in solid tumors has been limited due to tumor heterogeneity, immune suppression, and poor cellular infiltration. Emerging approaches involving localized CAR-T cell delivery, improved CAR design, and targeted antigen selection (such as HER2, MUC1, GD2, and B7-H3) are discussed as promising strategies to enhance therapeutic outcomes. Clinical studies highlighted in this review indicate improved local tumor control and potential to optimize surgical resections. Additionally, combining CAR-T therapy with surgery may reduce tumor recurrence and positively influence reconstructive outcomes. Overall, this review underscores CAR-T cell therapy as a potential adjunctive treatment in oncologic surgery, emphasizing the importance of interdisciplinary approaches to improve patient outcomes in solid tumor management.

## Graphical abstract

**Figure:**
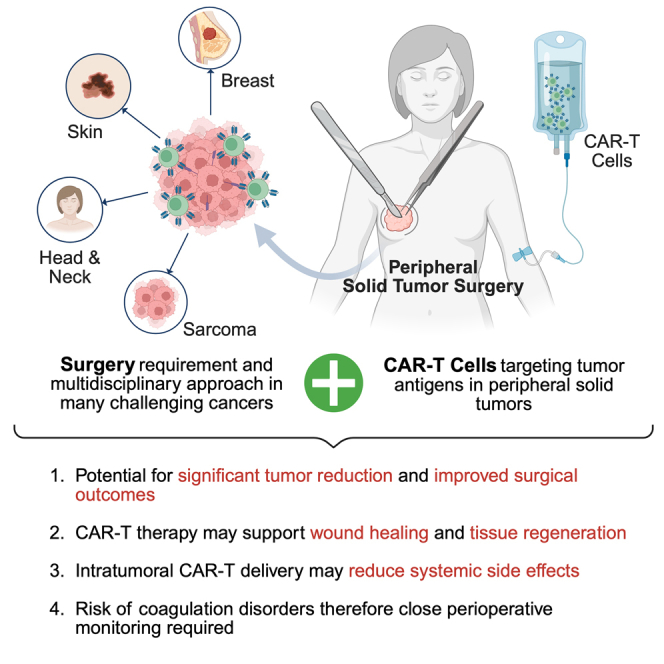

## Teaser

In this review, Knoedler et al. highlight the potential of integrating CAR-T cell therapy with reconstructive surgery for peripheral solid tumors. Combining advanced cellular immunotherapy with surgical approaches may enhance tumor control, reduce recurrence, and optimize reconstructive outcomes, underscoring the importance of interdisciplinary strategies in cancer treatment.

## Background

Cancer continues to be the second most common cause of death in the US totaling 1.9 million new cases and 609,360 deaths in the US in 2022.^1^ About 60% of US cancer patients undergo surgical cancer treatment.^2^ Peripheral solid tumors (PSTs), such as breast, malignant skin, head and neck cancers, and sarcomas represent challenges for patients and providers.^3^ They commonly require primary resection, the therapeutic gold standard for localized stages.^4^^,^^5^^,^^6^^,^^7^ PST resection necessitates reconstructive surgery to support patient rehabilitation and improve postoperative quality of life.^8^^,^^9^^,^^10^ Current cancer guidelines suggest a close coordination of oncologic resection and reconstructive surgery.^11^^,^^12^^,^^13^

In more advanced tumor stages, therapy may include systemic treatment options.^14^^,^^15^ Over the past decades, chemotherapeutics and radiotherapy have been established as standard (neo-)adjuvant therapy strategies. While such schemes have helped reduce cancer death rates, novel treatment concepts are needed to improve oncologic outcomes.^16^^,^^17^

The advent of chimeric antigen receptor (CAR)-T cells has provided an additional therapy line for patients with refractory or relapsed tumor burden.^18^ This seminal biotechnology involves specialized T cells that are engineered to target cancer cells based on their surface antigen signature. CAR-T cells have shown potent effects in hematological diseases, with their therapeutic potency in solid tumors being subject to ongoing translational efforts.^17^^,^^19^^,^^20^

The immunosuppressive tumor microenvironment (TME) in PSTs contains molecules that hinder CAR-T cell infiltration and effectiveness.^21^ Further, the administration of CAR-T cells can offset various side effects, ranging from cytokine release syndrome (CRS) to neurological toxicity and prolonged cytopenia.^22^ Knowledge of the strengths and limitations, safety, and side effects of CAR-T cells is paramount to leveraging systemic therapies with local surgical strategies.

However, there is a paucity of research work summarizing the scientific literature on CAR-T cells against PST and distilling practical implications for the reconstructive readership. Here, we aim to condense the current body of clinical trials and basic science on CAR-T cells for PST and outline the impact of CAR-T therapy on wound healing, functional and aesthetic recovery, and perioperative risk management. This line of research may offer valuable insights to reconstructive surgeons, enhancing their understanding of CAR-T cells and synchronize targeted anti-cancer efforts.

## CAR-T cells—From basic principles to clinical therapy

**Figure 1:**
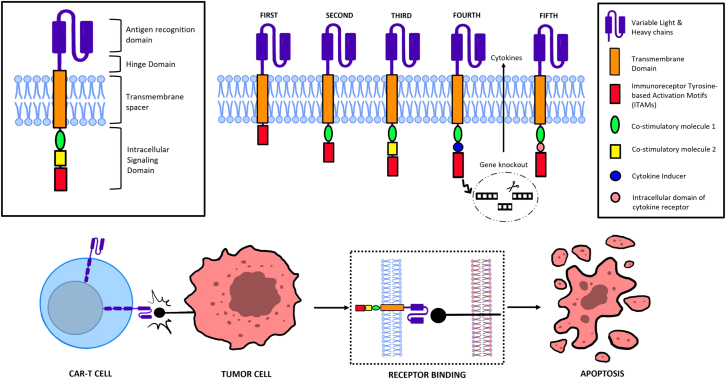
Evolution and mechanisms for CAR-T cell therapy First-generation CAR constructs contained a single CD3 ζ chain or FcεRIγ intracellular domain without costimulatory domains, whereas second-generation CARs integrated an additional cytoplasmic domain (e.g., CD28, 4-1BB, and OX-40) to expansion and cytokine production upon encountering a tumor antigen.^24^ Combining multiple costimulatory domains resulted in the third generation of CARs but did not further enhance the CAR-T cell efficacy.^25^^,^^26^^,^^27^ Therefore, fourth-generation CARs rediscovered the second-generation constructs with one single costimulatory domain. The fourth-generation CAR-T cells were engineered to release a transgenic cytokine (e.g., IL-12, IL-15, and IL-23) upon CAR signaling in order to modulate the tumorous immune regulation.^28^ The latest generation of CAR constructs leverages membrane receptors (e.g., IL-2 receptors) to promote pathway activation (e.g., Janus kinase [JAK]/signal transducer and activator of transcription [STAT] pathway) in an antigen-dependent manner and reduce the CAR’s off-target and off-tumor toxicity.^29^ Across the various generations, CAR-T cell-mediated antitumoral toxicity mainly relies on the activation of the intracellular signaling domains within the CAR-T cell, leading to the release of cytotoxic molecules such as perforin and granzymes, which induce apoptosis in the tumor cells.^23^

CAR-T cells are a form of cancer immunotherapy where T cells are genetically modified to express a specialized receptor, known as CAR. This receptor enables these T cells to specifically target and lyse cancer cells. Essentially, a CAR consists of three integral elements: an extracellular target-binding domain derived from antibodies that recognizes and binds to specific antigens on cancer cells; a transmembrane domain that anchors the CAR to the cell membrane; and an intracellular signaling domain that activates the T cell upon binding to the target antigen.^23^ The generations of CAR-T cells are in part distinguished by their intracellular signaling domains. To produce CAR-T cells, T cells are isolated from the patient (or healthy suitable donors) and then genetically engineered, often through viral vectors, to express the CAR. After being cultivated *ex vivo*, these are reintroduced into the patient (Figure 1).

## CAR-T cells in reconstructive surgery—From breast cancer to head and neck cancer

### CAR-T cells in breast cancer—from HER2 to c-Met

#### MUC1—*In vitro* and *in vivo* data

MUC1 is a transmembrane glycoprotein commonly found on epithelial cells.^30^ In tumor cells, the extracellular domain of MUC1 undergoes hypoglycosylation, leading to the generation of transformed MUC1 (tMUC1), which is overexpressed on >90% of triple negative breast cancers (TNBCs).^31^ Based on these findings, a CAR-T cell construct consisting of the signaling domains CD3ζ and CD28 and the antigen binding-site derived from the monoclonal antibody TAB004 against tMUC1 was designed.^32^ The MUC28z CAR-T cells demonstrated highly specific lysis of different tMUC1^+^ tumor models *in vitro*, while sparing healthy epithelial tissue breast organoids. Furthermore, the CAR-T cells showed significant upregulation of the leukocyte activation markers CD25 and CD11c as well as high levels of interferon (IFN)-*γ* and Granzyme B compared to control T cells and PBMCs. To evaluate the effectiveness of the CAR-T cells *in vivo*, cells of the tMUC1^+^ HCC70 tumor cell line were administered subcutaneously into NSG female mice, resulting in a significant reduction in tumor growth in the intervention group.^32^ The NCT04020575 phase 1/2 trial is a first-in-human study that aims to evaluate MUC1-targeting CAR-T cells in patients with metastatic breast cancer (BC). Study patients are assigned to three study arms (luminal, HER2^+^, and TNBC) to determine the safety and efficacy of MUC1-targeting CAR-T cells in these BC subtypes (NCT04025216).^33^

#### HER2—From bench to bedside

The receptor tyrosine kinase HER2 has emerged as another promising target antigen due to its overexpression in 30% of all breast tumor cells.^34^ In preclinical studies, HER2-targeting CAR-T cells led to regression of subcutaneously induced SKBR3 tumors, whereas tumor growth persisted in mice treated with untransduced T cells.^35^ Szöőr et al. studied the efficacy of CAR-T cells in HER2^+^ trastuzumab-resistant BC.^36^ The authors hypothesized that epitope masking and steric barriers through antibody binding by components of the extracellular matrix, including hyaluronan or MUC4, were the main reasons for the poor clinical response to trastuzumab. Therefore, different trastuzumab-resistant and HER2^+^ tumor cell lines (i.e., JIMT-1 and MDA-HER2.ffLUC) were injected subcutaneously into both flanks of NSG mice. The authors compared a control group of untreated mice to mice that were treated with 100 μg trastuzumab twice a week and to another group injected with a single dose of 5 × 10^6^ CAR-T cells on day 14 post-tumor transplantation. CAR-T cells were able to effectively eradicate tumor tissue, resulting in complete regression of tumor xenografts in the MDA-HER2.ffLUC model. Frozen sections of excised JIMT-1 tumors showed that HER2-directed CD8^+^ CAR-T cells could effectively penetrate trastuzumab-resistant tumors displaying a CCR7^−^CD45RA^−^ effector memory-like phenotype.^36^^,^^37^ An ongoing clinical study is using intraventricular injection of HER2-targeting CAR-T cells for the treatment of patients with different HER2^+^ tumors metastasized to the brain and/or meninges. Once a week, patients receive CAR-T cells for a total of three doses (NCT03696030).

### Additional CAR-T targets in breast cancer—From mesothelin to c-MET

Another BC target antigen is the surface protein mesothelin, which is overexpressed in 67% of TNBCs.^38^ This overexpression has been correlated with the development of resistance to chemotherapy and poor prognosis.^34^ A US phase 1 trial enrolled 186 patients to evaluate the safety and tolerability of mesothelin-targeting CAR-T cells in mesothelin^+^ metastatic BC. Before intravenous infusion of the CAR-T cells on day 0, premedication with acetaminophen and diphenhydramine and lymphodepletion with cyclophosphamide (day −7 to day −2) are performed (NCT02792114).

In certain cancer types, including BC, high receptor tyrosine kinase-like orphan receptor 1 (ROR1) expression levels have been reported, making it an interesting target for immunotherapies such as CAR-T cell therapy.^39^^,^^40^ A US phase 1 trial (NCT02706392) aimed to treat patients with ROR1^+^ cancers, including TNBC, with CAR-T cells. Following lymphodepleting chemotherapy, patients intravenously received CAR-T cells directed against the extracellular domain of ROR1. In four TNBC patients who had been treated with escalating doses of CAR-T cells, no severe toxicities were observed, while two patients experienced mild CRS. Ultimately, stable disease was observed in two patients at 15 and 19 weeks after CAR-T cell administration, respectively.^41^

**Figure 2:**
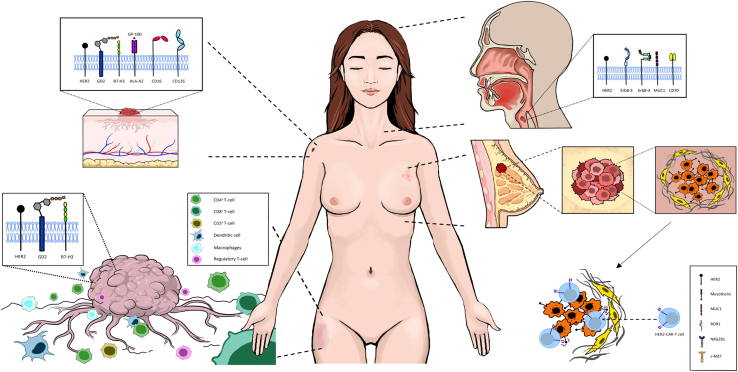
CAR-T cell therapies against different cancer entities Different CAR-T cell targets have been proposed for BC therapy ranging from HER2 over MUC1 and c-Met.^34^ While treatment with monoclonal antibodies, such as trastuzumab, or immune checkpoint inhibitors have reduced mortality rates and prolonged overall survival, further advancements are needed to overcome persisting therapy limitations (e.g., poor trafficking into BC tissue).^43^^,^^44^ CAR-T cell therapy carries the potential of enhanced tumor infiltration, which may lead to improved patient outcomes and enhanced therapy response.^34^ For skin cancer (SC), different targets have been explored in recent trials, and their basic molecular structures are illustrated. SC (especially malignant melanoma [MM]) cells have been shown to present HER2, GD2, B7-H3, CD16, and CD126 in various levels.^45^ CAR-T cells targeting these structures have presented promising results in preclinical models with clinical trials underway to scrutinize efficacy in patients with skin tumors.^45^^,^^46^^,^^47^^,^^48^^,^^49^ The variety of sarcoma subtypes and complex cell-cell/cell-molecule interactions in the tumor microenvironment involving dendritic cells, macrophages, or regulatory T cells represent persisting hurdles for CAR-T therapies. Antigens displayed on sarcoma cells are shown, as they stand as potential targets for CARs including HER2, GD2, or B7-H3.^50^,^48^^,^^51^ However, the variety of sarcoma subtypes complicates the identification of target structures expressed on different types of sarcoma.^52^ Moreover, further research is needed to establish CAR-T cells as another therapy pathway in head and neck cancer treatment. MUC1; CD70; and members of the ErbB receptor family, including HER2, ErbB-3, and ErbB-4, have been found overexpressed in HNSCC cells and have therefore been investigated as possible targets for CAR-T cell therapy.^53^^,^^54^^,^^55^^,^^56^^,^^57^^,^^58^ However, promising *in vitro* data remain to be translated into *in vivo* experiments to ultimately design clinical studies.

The receptor tyrosine kinase c-MET (c-MET) represents another potential target antigen, as it is overexpressed in approximately 50% of all BCs.^38^ Tchou et al. generated CAR-T cells with a c-MET-directed CAR.^42^ NSG mice were injected with c-MET-expressing SK-OV-3/luc tumor cells into the flanks, followed by intratumoral injections of c-MET-CAR-T cells or CD19 CAR-T cells. Bioluminescence measurement showed halted tumor growth. Following these promising *in vivo* results, an early clinical trial (NCT01837602) was conducted in six patients with metastatic BC. Participants received a single intratumoral injection of c-MET-directed CAR-T cells, which was well tolerated. Tumor resection was performed two days after CAR-T cell administration. In addition to a high number of CD4^+^ T cells, recruitment of CD68^+^ macrophages into the necrotic tumor tissue was observed^42^ (Figure 2).

### CAR-T cell therapy in skin cancer—Targets and approaches

#### GD2—Translational findings

GD2 is a ganglioside subtype that is overexpressed in various malignancies, including melanoma.^59^ In mice receiving tumor cells and CAR-GD2 T cell therapy, 80% of the mice receiving CAR-GD2 T cell therapy survived over 100 days, demonstrating a significant survival benefit.^60^

In pediatric and young adult patients with GD2^+^ solid tumors, anti-GD2-CAR-T cell treatment is being explored in a phase 1 trial. A vector equipped with a “suicide switch” including the caspase dimerization domain ICD9 is added as a safety measure to cause the death of the engineered T cells in case of unexpected toxicity (NCT02107963). A 3^rd^ generation CAR against GD2 comprising CD28 and OX-40 costimulatory domains demonstrated efficacy in preclinical *in vitro* and *in vivo* models, resulting in a phase 1 clinical trial for pediatric and young adult patients with GD2^high^ tumors.^61^

#### B7-H3—A promising target protein?

B7 homolog 3 protein (B7-H3), also known as CD276, is overexpressed in different types of cancer cells, including malignant melanoma (MM).^62^ Aung et al. investigated B7-H3 expression and cluster of differentiation 31 (CD31), also known as platelet endothelial cell adhesion molecule (PECAM-1), in Merkel cell carcinoma (MCC). They stated that coexistent expression of B7-H3/CD31 is an indicator of unfavorable outcomes. In primary MCC, higher levels of colocalized expression of B7-H3 and CD31 significantly correlated with greater depth of invasion and beyond increased tumor size and increased vascular density.^63^ Tang et al. conducted the first-in-human study on CAR-T cells targeting B7-H3 for the treatment of recurrent anaplastic meningioma. Their findings demonstrated that CAR-T cells targeting B7-H3 could inhibit tumor growth without causing off-tumor toxicity.^62^^,^^64^ An ongoing clinical study is designed to investigate anti-B7-H3 CAR-T cells in patients with advanced B7-H3^+^ solid tumors (NCT04691713).

### Additional CAR-T targets in skin cancer therapy—From CD16 to HER2

CD16 is a low-affinity immunoglobulin G Fc receptor, persistently expressed by human natural killer cells (NK cells) and polymorphonuclear neutrophils.^46^ Therefore, CD16 has been investigated by looking at NK cells to improve the efficacy of immunotherapy in MM. Lee at el. scrutinized CD16 and NK cell functions, undermining the versatile function of NK cells and suggesting the investigation of possible CAR-NK cells for the treatment of solid tumors.^65^^,^^66^ For a variant of CAR-T cells targeting CD16, elevated cytotoxicity against melanoma cells was observed *in vitro*, alongside wild-type antibodies against CD20 or MCSP. The presence of glycoengineered antibodies could enhance the performance of any variant of CD16-CAR-T cells. Antitumor activity of CD16 targeting CAR-T cells was investigated with melanoma cells, but also with pancreatic cancer cells and Raji lymphoma cells.^47^

High glycoprotein 100 (gp100) is a common feature of MM.^67^ Recently, CAR-T cells with a 4-1BB costimulatory domain and extracellular innate immune engagers have been designed targeting the gp100/major histocompatibility complex (MHC) complex. Autologous T cells, once reintroduced into the patient, not only recognize and destroy cells expressing gp100 peptides in the MHC class I complex but also stimulate the innate immune system to further improve cancer rejection. This approach is being investigated in a clinical trial (NCT03649529).^66^^,^^68^ While HER2 is considered a mainstay target in BC treatment (for details on HER2, please see the breast cancer chapter), recent research efforts aimed to elucidate its role in skin cancer (SC). Melanoma cells expressing HER2 could be eliminated with HER2 CAR-T cells *in vitro* and *in vivo*. Curative effects *in vivo*, however, could only be detected in xenograft developed in a mouse strain, transgenic for human interleukin (IL)-2 and known as non-obese diabetic (NOD)/severe combined immunodeficiency (SCID) IL-2 receptor gamma knockout^45^ (Figure 2).

### CAR-T cells in sarcoma therapy—Potent therapeutics?

#### HER2—More than a breast cancer target antigen?

Expression of HER2 can represent a negative prognostic factor present in bone and soft tissue sarcoma. Of note, due to the lack of gene amplification, the HER2 expression level on sarcomas is significantly lower compared to other HER2^+^ solid tumors, rendering HER2-targeting therapies more challenging.^69^ Ahmed et al. performed a study of 19 pediatric and adult patients with refractory or recurrent metastatic HER2^+^ sarcoma, using a HER2-CD28^−^CD3ζ second-generation CAR. Stable disease was observed in four patients, whereas the residual tumor could be fully resected in three other patients. One of these patients had a cellular necrosis rate of more than 90% in the tumor, demonstrating the high potency of the administered CAR-T cells. In addition, HER2 CAR-T cells were detected in tissue samples of surgically removed tumors, highlighting tumor tissue infiltration and persistence. CAR-T cells persisted up to 18 months.^70^ The study was continued thereafter, with ten patients aged between 4 and 54 years with refractory/metastatic HER2^+^ sarcoma participating. CAR-T cells could be detected in all patients at 6 weeks post-infusion, and expansion occurred in most patients. One osteosarcoma patient with lung metastasis had an ongoing complete remission for 32 months, while three patients had stable disease. In five participants, progressive disease was observed.^71^^,^^72^

#### GD2—A specific target for potent sarcoma therapy?

GD2 is highly expressed on certain sarcomas such as osteosarcoma, Ewing sarcoma, and rhabdomyosarcoma.^50^ Long et al. designed a third-generation GD2-CAR and demonstrated its potency in lysing GD2^+^ sarcoma cells *in vitro.*^69^ Yet, this promising preliminary data could not be replicated in a murine xenograft model due to the accumulation of monocyte-derived suppressor cells, which are known to hamper CAR-T cells.^73^ Nevertheless, under simultaneous administration of immunomodulatory drug all-trans retinoic acid, the efficacy of CAR-T cells could be restored.^74^ Chulanetra et al. also investigated the potential of GD2-targeting CAR-T cells by combining CAR-T cells with subtoxic doses of the chemotherapy drug doxorubicin, resembling the clinically established approach of metronomic immunomodulatory chemotherapy. A fourth-generation CAR incorporating a CD28 transmembrane and cytoplasmic domain, a 4-1BB intracellular TNF receptor-associated factor (TRAF)-binding domain, a CD3ζ intracellular domain, and an inducible self-destructive caspase-9 genetic cassette was used. Results demonstrated that GD2-CAR-T cells effectively killed GD2^+^ osteosarcoma cells *in vitro*. The researchers further observed that GD2-CAR-T cells led to an increase in PD-L1 expression on both osteosarcoma and GD2-CAR-T cells. Importantly, when low doses of doxorubicin were combined with GD2-CAR-T cells, the killing of osteosarcoma cells was significantly enhanced.^75^

A US phase 1 trial aims to investigate dosing and antitumor responses of anti-GD2-CAR-T cells in GD2^+^ cancers, including osteosarcoma, Ewing sarcoma, and rhabdomyosarcoma, that have either relapsed after initial treatment or did not respond to conventional therapies at all. Interestingly, the researchers plan to insert another gene (*C7R*) into the GD2-targeting CAR-T cells, which will provide a constant cytokine supply to help the cells persist in the blood to address the suggested mechanism of failure in the previous study (NCT03635632).

#### B7-H3—Translational findings

Another promising target antigen, B7-H3, is a transmembrane glycoprotein that, similar to GD2, is specifically overexpressed in sarcoma.^50^ Majzner et al. tested anti-B7-H3 CAR-T cells on osteosarcoma and Ewing sarcoma xenografts in a mouse model. The CAR construct included various signaling domains, such as a 4-1BB costimulatory motif and a CD3ζ signaling domain. The study conducted *in vivo* experiments using immunodeficient mice with different tumor models, including osteosarcoma and Ewing sarcoma. In both types of sarcomas, complete regression and eradication of tumors were achieved after infusion of CAR-T cells, leading to a significantly longer survival of the mice compared to the untreated controls. However, the authors also highlighted the importance of antigen density for CAR-T cell efficacy. When testing against Nalm-6 leukemia cells that expressed different levels of the target antigen, it was observed that anti-B7-H3 CAR-T cells showed varying levels of killing and activation dependent on the density of the target antigen. Lower B7-H3 expression on cancer cells resulted in decreased CAR-T cell activity. This finding emphasized the need to determine the optimal antigen density for successful CAR-T cell therapy.^76^

A phase 1 US trial is testing B7-H3-directed CAR-T cells in patients younger than 21 years old with relapsed or refractory B7-H3^+^ tumors, including different sarcoma entities. Following lymphoablative chemotherapy with fludarabine and cyclophosphamide, participants receive a single intravenous injection of CAR-T cells at four different doses (from 3 × 10^5^/kg to 1 × 10^7^/kg) (NCT04897321) (Figure 2).

### CAR-T cells and head and neck cancer

Current research on CAR-T cell treatment for head and neck squamous cell carcinoma (HNSCC) is mainly based on the preclinical stage. The transition from preclinical studies to clinical trials is warranted but still faces challenges ranging from limited trafficking and penetration into the tumor site and immunosuppressive TME to exhaustion of CAR-T cells, severe side effects, and tumor heterogenicity.^16^^,^^53^^,^^54^

#### MUC1—From bench to bedside

MUC1 is overexpressed in HNSCC.^55^ MUC1 expression can be increased by the addition of exogenous human IL-22 recombinant protein, enhancing the functionality of T cells. Examining the validated antitumor activity in MUC1-targeting CAR-T cells, and in CAR-MUC1-IL-22-T cells secreting IL-22, CAR-MUC1-IL-22-T cells have exhibited higher cytotoxicity against MUC1^+^HNSCC cells. Interestingly, 72 h after the introduction of IL-22 cytokine, the peak MUC1 expression was attained. Therefore, IL-22-related experiments are commonly conducted at the 72-h mark to evaluate tumor cell apoptosis. To validate the *in vitro*-observed effectiveness of CAR-T cells, an NOD/SCID mouse model for *in vivo* studies has been employed, demonstrating anti-cancer efficacy and superiority of CAR-MUC1-IL-22-T cells over non-cytokine armed CAR-T cells.^56^

#### CD70—A clinical target for CAR-T cell therapy in head and neck cancer?

CD70 stands as the only established ligand for CD27. CD27 is a member of the tumor necrosis factor (TNF) receptor superfamily, with CD70 belonging to the associated ligand superfamily. CD70 and CD27 exhibit abnormal expression in various hematological and solid malignancies, contributing to tumor progression and immunosuppression within the TME.^77^ Park et al. investigated the mRNA expression of 18 reported general CAR-T cell targets in HNSCC to detect possible CAR-T cell target antigens for HNSCC therapy. Out of the potential cell surface protein targets screened, nine candidates exhibited a significant elevation in mRNA expression in HNSCC cells compared to the control group. CD70 overexpression was limited to specific HNSCC cancer subtypes such as laryngeal, oral cavity, and tongue cancers. Also, when compared to corresponding adjacent healthy tissues, considerable variability was observed in CD70 expression among 22 individual cancer cases. Interestingly, CD70 expression in HNSCC tumor cells is correlated with tumor differentiation and is also found in tumor-associated blasts and endothelial cells.^78^ Still, anti-CD70 CAR-T cells demonstrated remarkable *in vitro* efficacy against CD70 overexpression cell lines; however, no further *in vivo* research was conducted.^53^

### ErbB receptor family—A group of promising targets?

The ErbB receptor family includes specific transmembrane tyrosine kinase proteins: epidermal growth factor receptor, also known as ErbB1; ErbB2, also known as HER2; ErbB-3; and ErbB-4, all of which are overexpressed to varying degrees in human cancer including HNSCC.^79^^,^^80^^,^^81^ While targeting individual ErbB receptors can have anti-cancer effects, its efficacy is often impaired due to the development of resistance, which is based on the increasing activity of other receptors of the ErbB family. Therefore, Davies et al. developed genetically engineered T cells for flexible targeting of ErbB dimers.^82^^,^^83^^,^^84^ These T cells, enhanced with the T1E28z CAR, are selectively triggered by all forms of ErbB1-based homo- and heterodimers. Thereby, they can eliminate a wide range of tumor cell lines originating from various tumor types, with antitumor activity observed in mice bearing xenografts, characterized by ErbB1/2 or ErbB2/3 overexpression.^83^ Consequently, T1E28z is being investigated in CAR-T cell treatment for HNSCC. It is implemented in the so-called T4 immunotherapy, an autologous cell therapy, where peripheral blood T cells are genetically modified using a retroviral vector to simultaneously express two chimeric receptors, T1E28z and a cytokine co-receptor (4αβ) enabling improved CAR-T cell expansion. Van Schwalkwyk et al. conducted a phase 1 clinical trial to test T4 immunotherapy with intratumoral application in HNSCC for efficacy and toxicity.^85^ All treatment-related adverse effects were mild (grade II or less). Disease stabilization was observed in 60% of the subjects 6 weeks after CAR-T cell application (in contrast to the fast progression at the beginning of the trial). This encourages the intratumoral application and general introduction of T4 immunotherapy in HNSCC CAR-T cell therapy.^57^ Application of T4^+^ T cells *in vivo*, whether intravenously or directly into the tumor, resulted in partial tumor reduction without clinical or histopathological toxicity observed.^86^ Further, when investigated *in vivo*, intratumoral administration of T4^+^ T cells led to disease regression. Moreover, the T cells remained at the injection site for several days without causing CRS or other side effects.^57^^,^^85^^,^^87^

### HER2—*In vitro* and *in vivo* head and neck cancer studies

It has been reported that an increase in HER2 overexpression is linked to poorer prognosis and higher recurrence rates in HNSCC.^54^^,^^88^^,^^89^ A challenge faced by CAR-T cell therapy in solid tumors is the immunosuppressive TME. By producing immune checkpoint ligands that interfere with co-stimulation, and by counteracting pro-inflammatory cytokines, the tumor environment inhibits the sustained activation of effector T cells.^84^^,^^90^^,^^91^ Therefore, Rosewell Shaw et al. proposed a novel approach for the amplification of the antitumor response of HER2-specific CAR-T cells by pretreating with an oncolytic adenovirus (CAd), which triggers local oncolysis and induces the expression of immune-stimulating molecules. They revealed that IL-12p70, a cytokine derived from adenovirus, promotes persistence of anti-HER2 CAR-T cells within the tumor site and eradication of disseminated HNSCC cells *in vivo.*^91^^,^^92^ A first-in-human phase 1 study of binary oncolytic adenovirus in combination with HER2-specific autologous CAR-T cells in patients with advanced HER2^+^ solid tumors is designed to investigate this treatment approach. Both CAd vector targeting CAR-T cells and HER2-targeting CAR-T cells are research compounds. A single intratumoral injection of CAd vector CAR-T cells will be injected into an appropriate tumor site. Separately, autologous anti-HER2 CAR-T cells, created from the patient’s blood will be applied (NCT03740256) (Figure 2).

## Practical implications for reconstructive surgeons

With more and more PST patients receiving CAR-T therapy, the main barriers for synergistic surgical and systemic therapy include (1) the effects of CAR-T cells on wound and tissue healing, (2) the impact of CAR-T cells on functional/aesthetic recovery, (3) coagulation disorders/bleedings under CAR-T cell therapy, and (4) the concept of local and locoregional CAR-T therapy. While these remain to be investigated for CAR-T against PST, research on CAR-T therapy for other oncological and non-oncological may provide preliminary insights.

### The effects of CAR-T cells on wound healing and tissue recovery

Wound healing is the cornerstone of reconstructive surgery. While CAR-T therapy (especially anti-CD19 CARs) has been implicated with severe dermatological complications (e.g., maculopapular eruption, erythematous rash, and bullous eruption), to date, there is no literature available that links CAR-T therapy to wound healing disorders.^93^^,^^94^ However, there is emerging evidence that elucidates the wound healing-supporting effects of CAR-T cells. For example, Moradi et al. introduced the concept of CAR-T therapy to burn care.^95^ Previous work has highlighted that burns led to increased levels of HIF1α that alters antigen presentation and T cell stimulation. In more detail, HIF1α impairs function of CD11^+^ dendritic cells by reducing the expression of MHC class I and II molecules, paramount for effective antigen presentation.^96^ Additionally, CD8^+^ cytotoxic T lymphocytes exhibit reduced proliferation and effector function under high HIF1α levels.^97^ Conversely, CD4^+^CD25^+^FoxP3^+^ T_regs_ have been shown to become more resistant to BAX- and BAK-mediated mitochondrial apoptosis, fueling an immunosuppressive microenvironment.^98^ The immunosuppressive microenvironment has been implicated with poor pathogen clearance, prolonged inflammation, and excessive scarring in burn wounds.^99^ Thus, Moradi et al. proposed to generate CAR-T cells that turn the immunosuppressive milieu upside-down.^95^ For example, CAR-T could be engineered to secrete pro-inflammatory cytokines (e.g., IL-12, IL-15, or IFN-γ).^100^

In a broader context, CAR-T cells have also been investigated to target a persisting bottleneck in wound healing. Pathological fibrosis has been identified as a main driver of hypertrophic scarring and dysfunctional tissue via long-term activation of transforming growth factor β.^101^ Thus, a 2022 US study generated transient antifibrotic CAR-T cells *in vivo* by delivering mRNA in CD5-targeting lipid nanoparticles. CAR-T cells were directed against fibroblast activation protein (FAP), a marker of activated fibroblasts, and evaluated in a mouse model of heart failure. Efficient delivery of modified mRNA encoding the CAR to T lymphocytes was observed, which produced transient, effective CAR-T cells *in vivo*. Anti-FAP CAR-T cells exhibited trogocytosis (i.e., a process where CAR-T cells aquire membrane components from target cells, enhancing their activation, persistence, and cytotoxicity) and retained the target antigen as they accumulated in the spleen.^102^ Treatment with modified mRNA-targeted LNPs reduced fibrosis, restored cardiac function after injury, and balanced the heart-weight-to-body-weight ratio (i.e., a measure of cardiac hypertrophy).^103^ These insights echoed previous findings that demonstrated a significant reduction in cardiac fibrosis in mice that had been treated with the anti-FAP CAR-T cells compared to controls at 8 weeks post-injection. Prior to CAR-T therapy, mice were administered AngII/PE, known to induce severe cardiac fibrosis and dysfunction by increasing afterload and chronotropy.^104^ Here, the authors also observed a partial rescue of both systolic and diastolic cardiac function in mice treated with CAR-T cells.^105^

Collectively, reconstructive surgeons may carefully check on the patient’s specific CAR-T treatment and recent updates on its impact on wound healing. To date, current literature points toward a positive, if any, effect of CAR-T therapy on wound healing.

### The potential of CAR-T cells to improve functional and aesthetic outcomes

The extent of tissue resection required to achieve tumor-free margins in PST depends on several factors (e.g., depth of invasion, locoregional spread, and intrinsic neoplastic potential).^106^ Similarly, the aesthetic and functional outcomes of reconstructive efforts are linked to the anatomical subunits and tissues affected by the malignancy. For example, reconstruction of important subunits in the face (eyelids, nose, and lips) is challenging.^107^ To preserve as much healthy tissue as possible, CAR-T cell therapy could pose as a novel adjuvant or neoadjuvant treatment option. In fact, targeted anti-mesothelin T cell therapy has demonstrated the ability to achieve significant tumor shrinkage of up to 80% in 32 patients with refractory mesothelin-expressing solid tumors.^108^ Achieving preoperative tumor shrinkage may reduce the amount of tissue that needs to be removed for tumor clearance and help preserve aesthetic and function. Unlike chemotherapy and radiation, there are currently no reports suggesting that CAR-T cell therapy causes fibrosis or permanent changes to native tissue quality.^109^^,^^110^ Uslu et al. utilized mesothelin-specific CAR-T cells (CARM5) in both TNBC and pancreatic ductal adenocarcinoma xenograft models.^111^ The authors demonstrated that locally applied CAR-T cells (in a fibrin glue-based carrier) were successful at clearing residual cancer cells after incomplete surgical resection without impairing local wound healing. This, in fact, demonstrates that CAR-T cells could have the potential to serve as an adjunctive treatment to clear incompletely resected malignant cells. Particularly, in head and neck cancers, where achieving clear margins often necessitates extensive resections in cosmetically and functionally sensitive regions, a topical CAR-T cell delivery strategy could help avoid overtreatment and may allow preservation of critical structures at the same time. However, it is noteworthy that these preclinical findings remain to be translated and corroborated in clinical studies before reigniting the discussion of modified margin-free tumor resection.

The aesthetic and functional outcomes of patients undergoing tumor resection of the face, for example, are generally poor.^112^ Local tissue re-arrangement and free flap-based reconstruction are able to achieve wound closure, but the near-normal reconstruction of intricate anatomical subunits such as the eyelids, nose, and lips is not possible with conventional reconstructive techniques.^113^ Vascularized composite allotransplantation (VCA), such as face or limb transplants, has emerged as a novel reconstructive option and is able to restore both form and function.^114^ However, there is limited indication and experience for the use of VCAs in patients with history of malignancy given the necessity of immunosuppression, which places the patient at increased risk of recurrence and *de novo* cancer occurrence, and thus presents a relative contraindication to proceed with transplant.^115^ In this context, the implementation of CAR-T cell therapy may improve curative rates and locoregional control and pave the way to safely performing VCAs in this population. Furthermore, CAR-T cell therapy has also been explored as a novel immunosuppressive approach with the goal to limit the need for systemically toxic standard immunosuppression such as tacrolimus.^116^ Experimentally, donor human leukocyte antigen-specific CAR-T_regs_ cells have been used to suppress alloreactivity in preclinical murine models.^117^^,^^118^^,^^119^ In conclusion, the integration of CAR-T cell therapy in reconstructive surgery could potentially transform treatment options for the patients with invasive malignancies by improving locoregional tumor control and shifting the risk-benefit profile of VCAs and immunosuppression toward a more favorable outcome. This in turn could improve the aesthetic and functional outcome in this cohort.

A critical functional consideration of CAR-T cell therapy is its impact on nerve regeneration. Neurotoxicity is a well-documented side effect, with a broad spectrum of neurological changes reported in patients treated with CAR-T cells. For example, in a cohort of patients receiving anti-CD19 CAR-T cells, nearly 40% experienced symptoms of neurotoxicity.^120^ These ranged from dysesthesias, focal weakness, and suspected peripheral neuropathy to more global neurological effects, including aphasia and cognitive impairment.^121^ Conversely, emerging research in preclinical models has demonstrated that engineered transiently self-reactive T cells can offer neuroprotection in the context of central nervous system injuries by modulating myeloid cells via IFN-γ.^122^ This protective effect suggests a potential future application in reconstructive surgery, where enhancing nerve regeneration and functional recovery could improve reconstructive outcomes. However, further dedicated studies are necessary to elucidate how CAR-T cell therapy impacts nerve regeneration and how it might be harnessed to modulate nerve regeneration and support neurologic recovery in the reconstructive setting. This is particularly relevant as CAR-T cells could persist long-term, even after reconstructive effects have been concluded. In fact, previous studies demonstrated that CD19-targeting CAR-T cells (CTL019) persisted for over 4 years in patients achieving complete remission.^123^

Overall, more dedicated research is warranted to pinpoint the effects of CAR-T cells on functional and aesthetic recovery. To date, the literature suggests that CAR-T cells hold promise for novel (neo-)adjuvant protocols and improved outcomes of advanced reconstructive techniques. However, reconstructive surgeons should consider the risk of impaired neurological recovery.

### Coagulation disorders and bleedings under CAR-T therapy

Perioperative bleeding remains a major concern in tumor patients undergoing surgery.^124^ Bleeding poses a significant concern in CAR-T patients. There are different hypotheses that link CAR-T therapy to bleeding events and coagulation disorders, including the disruption of the endothelial barrier through the activation of CD31^+^ endothelial cells via IL-6, TNF-α, and IFN-γ during CRS, as well as thrombocytopenia and impaired hepatic synthesis of clotting factors such as fibrinogen, factor V, and factor VIII.^125^ A 2020 Chinese analysis of 100 patients with relapsed and refractory hematological malignancies reported high incidences of coagulation disorders, including elevated D-dimer in 50% and increased levels of fibrinogen degradation product in 45% of patients, respectively. The majority of these (73%) recovered without any medical treatment. Coagulation disorders commonly peaked during between 6 to 20 days after CAR-T cell infusion paralleling previous findings by Mei et al.^126^^,^^127^ While there are additional articles that have proposed similar incidences of bleeding events, a recent meta-analysis of 7,040 CAR-T patients calculated more moderate rates with pooled incidences of any bleeding events per patient-month standing at 1.9%.^128^ Notably, the large majority of articles focused on hematological diseases without delving into CAR-T therapy for solid tumors, let alone PST.^129^

Focusing on solid tumors and PST, research on coagulation disorders and bleeding events is scarce. In a patient-derived murine colon carcinoma xenograft model of anti-HER2 CAR-T cells, the authors reported complete tumor eradication without any signs of bleeding/coagulation disorders.^130^ In an ongoing phase 1 study, eleven patients with advanced pancreatic and biliary tract cancers were infused up to two cycles of 2.1 × 10^6^/kg anti-HER2 CAR-T cells. Here, preliminary findings reported one case of upper gastrointestinal bleeding (NCT01935843).^131^ While the articles did not cover any PST, HER2 is considered a promising target in PST, as previously described.

Collectively, the advent of CAR-T therapy for PST calls for future research on the risk of bleeding/coagulation disorders in this patient cohort. Current research stemming from hematological malignancies points toward a considerable risk of such events, especially within the first 6 to 20 days following CAR-T treatment. If possible, reconstructive surgeons may postpone any surgical reconstruction to a later time point, thoroughly evaluate coagulation markers preoperatively, and closely manage patients postoperatively.

### Local and locoregional CAR-T therapy

Surgical interventions are predestinated for local therapies. While systemic CAR-T therapy is considered the current gold standard, recent research has investigated the local administration of CAR-T therapy. For instance, Brown et al. demonstrated the safety and high bioactivity of local anti-IL13Ra2 CAR-T cell administration into the resection cavity of three patients with glioblastoma or intralesional delivery into multifocal glioblastoma. The authors reported that CAR-T therapy was well tolerated with 2 patients experiencing transient anti-glioma responses.^132^ The same group also reported a case report of a single patient with recurrent multifocal glioblastoma who received multiple local infusions of CAR-T cells targeting IL13Ra2, concluding that administration into the resected cavity controlled local relapse and progression of glioblastoma even in non-injection sites.^133^ A recent clinical trial investigated c-Met-targeting CAR-T cells that were injected into four patients with metastatic BC with accessible cutaneous or lymph node metastases at single doses of up to 3 × 10^8^ cells per patient. AR T mRNA was detectable in the peripheral blood and injection in two patients. The authors further found that such intratumoral injection was well tolerated, triggered local inflammatory responses within tumors, and navigated macrophages to the injection site.^42^ Recently, Uslu et al. tested the feasibility of locally administered anti-mesothelin CAR-T cells in two partial resection xenograft models using the MDA-MB-231 cell line (i.e., TNBC cell line). The authors demonstrated that the local delivery of CAR-T cells was effective in clearing residual cancer cells after incomplete surgical removal. This adjuvant approach led to significantly longer overall survival in immunocompetent C57BL/6 mice without signs of impaired wound healing or coagulation.^111^ Another study integrated CAR-T cells targeting the human chondroitin sulfate proteoglycan 4 into hyaluronic acid hydrogel. The authors injected the hydrogel into the tumor cavity of mice, where it inhibited the local recurrence and distant spreading of melanomas. The hydrogel, which functioned as a reservoir, promoted equal distribution of the CAR-T cells within the surgical bed.^134^

Overall, current research underpins the potential potency of local CAR-T therapy. While the optimal time point of perioperative CAR-T administration remains to be determined, reconstructive surgeons may consider this treatment strategy and consult with the treating oncologists to weigh the strengths and limitations of local (and intraoperative) CAR-T administration (Table 1).

**Table 1:** Implications of CAR-T therapy for reconstructive surgeons

Category	Implications for reconstructive surgeons
Wound healing & tissue recovery	• CAR-T therapy has been linked to severe dermatological complications, but no direct evidence suggests impaired wound healing. • Emerging research suggests potential benefits, such as immunomodulation and reduced fibrosis for improved healing. • Need for careful patient monitoring and review of emerging literature.
Functional & aesthetic outcomes	• Potential for CAR-T therapy as a neoadjuvant/adjuvant to reduce tumor burden and preserve healthy tissue. • Unlike chemotherapy/radiation, CAR-T does not appear to cause fibrosis or permanent tissue damage. • Potential role in vascularized composite allotransplantation (VCA) by improving tumor control and reducing reliance on toxic immunosuppressants. • Possible neurotoxicity may impair nerve regeneration; further research is needed.
Coagulation disorders & bleeding risks	• CAR-T therapy has been associated with coagulation disorders and bleeding risks, particularly during the first 6–20 days post-infusion. • Elevated D-dimer and fibrinogen degradation products have been observed in patients. • Consider delaying reconstructive surgery if possible, monitoring coagulation markers preoperatively, and closely managing postoperative risks.
Local & locoregional CAR-T therapy	• Research supports local CAR-T administration, which may enhance surgical outcomes and tumor control. • Intratumoral or cavity-injected CAR-T cells have shown promise in glioblastoma, breast cancer, and melanoma models. • Local administration may reduce systemic toxicity while preserving antitumor efficacy. • Close collaboration with oncologists is recommended to assess feasibility and timing of perioperative CAR-T application.

Reconstructive surgeons should consider different implications when treating patients who have received CAR-T therapy. Such implications include wound healing/tissue recovery, functional/aesthetic outcomes, risk of coagulation disorders/bleeding, and local/locoregional CAR-T Therapy. CAR-T, chimeric antigen receptor T cell therapy; VCA, vascularized composite allotransplantation.

## Conclusion

PSTs remain a persisting challenge calling for coordinated and multidisciplinary strategies. CAR-T cells have emerged as a novel therapy against PSTs.^135^ As the research on CAR-T cells progresses and expands beyond hematological malignancies onto solid tumors, the complexity of therapies may perplex healthcare providers and complicate joint efforts. Therefore, up-to-date knowledge of the strengths and limitations of CAR-T cells is paramount to leverage surgical and non-surgical treatment modalities, and regional CAR-T cell delivery is increasingly tested to improve surgical and non-surgical interventions. Overviewing the current literature with focus on wound healing, functional/aesthetic recovery, and perioperative patient management carries the potential to synchronize systemic and surgical therapies to improve PST outcomes. Ultimately, this line of research may supercharge CAR-T therapy against PST.

## Acknowledgments

The authors received no financial support for the research, authorship, and publication of this article.

## Author contributions

L.K. conceptualized the review, led the coordination of research efforts, and contributed to manuscript drafting. K.H. and D.A.S. offered clinical insights and critically revised the manuscript for important intellectual content. F.D., J.C., B.E., and M.S. assisted with data collection and interpretation and edited the manuscript drafts. B.-S.K., M.A., T.S., J.I., A.G.L., M.J., and S.K. provided substantial contributions to the discussion of the content and reviewed the manuscript critically for intellectual content. A.C., M.R., M.H., H.P., M.P., B.P., and M.K.-N. contributed to the conception of the work and revised it critically for important intellectual perspectives.

## Declaration of interests

M.R. holds patents related to CD19 CAR-T cells; served as a consultant for NanoString, Bristol Myers Squibb, GlaxoSmithKline, Scailyte, Bayer, and AbClon; and receives research funding from AbClon, NanoString, Oxford Nanoimaging, viTToria Biotherapeutics, CURIOX, and Beckman Coulter. M.R. is the scientific founder of viTToria Biotherapeutics. M.P. received speaker’s honoraria and travel funds from Amgen, Kite, and Takeda.
